# Publisher Correction: Oxygen respiration and polysaccharide degradation by a sulfate-reducing acidobacterium

**DOI:** 10.1038/s41467-023-43998-2

**Published:** 2023-12-01

**Authors:** Stefan Dyksma, Michael Pester

**Affiliations:** 1 Leibniz Institute DSMZ—German Collection of Microorganisms and Cell Cultures, Department of Microorganisms, Braunschweig, Germany; 2 Technical University of Braunschweig, Institute of Microbiology, Braunschweig, Germany

**Keywords:** Soil microbiology, Bacterial physiology, Element cycles, Microbial ecology

Correction to: *Nature Communications* 10.1038/s41467-023-42074-z, published online 10 October 2023

The original version of this Article contained an error in Fig. 4b, in which some of the labels indicating normalized transcriptional activity were inadvertently placed in incorrect positions. This has been corrected in both the PDF and HTML versions of the Article.

