# Supplementary material for: Oxygen respiration and polysaccharide degradation by a sulfate-reducing acidobacterium
Source: Nat Commun. 2023 Oct 10;14:6337. doi: 10.1038/s41467-023-42074-z (PMC10564751; doi:10.1038/s41467-023-42074-z)
Supplement: Supplementary file 1 — Supplementary Information [file 41467_2023_42074_MOESM1_ESM.pdf]

# Supplementary Information

**Oxygen respiration and polysaccharide degradation by a sulfate-reducing Acidobacterium**

Stefan Dyksma*[,1] and Michael Pester*[1,2]

1 - Leibniz Institute DSMZ – German Collection of Microorganisms and Cell Cultures, Department of Microorganisms, Braunschweig, Germany
2 - Technical University of Braunschweig, Institute of Microbiology, Braunschweig, Germany

* To whom correspondence should be addressed: Leibniz Institute DSMZ – German Collection of Microorganisms and Cell Cultures, Department of Microorganisms, Braunschweig, Germany Tel +49 531 2616420, stefan.dyksma@dsmz.de; Tel +49 531 2616327, michael.pester@dsmz.de

**Supplementary Figures S1-S5**

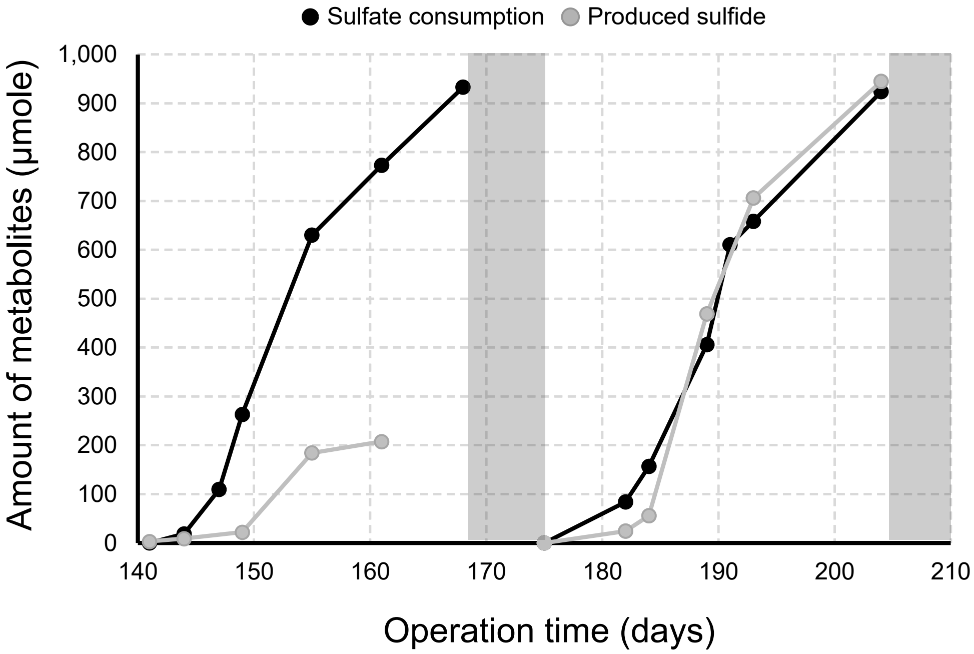


**Supplementary Figure S1.** Consumed sulfate and produced sulfide in the anoxic periods between days 141-161 and days 175-204. Oxic periods are indicated by grey areas. The produced sulfide accounted only for a fraction of the reduced sulfate between day 141 and day 161. Data on produced sulfide is not available for day 168. Source data are provided as a Source Data file.

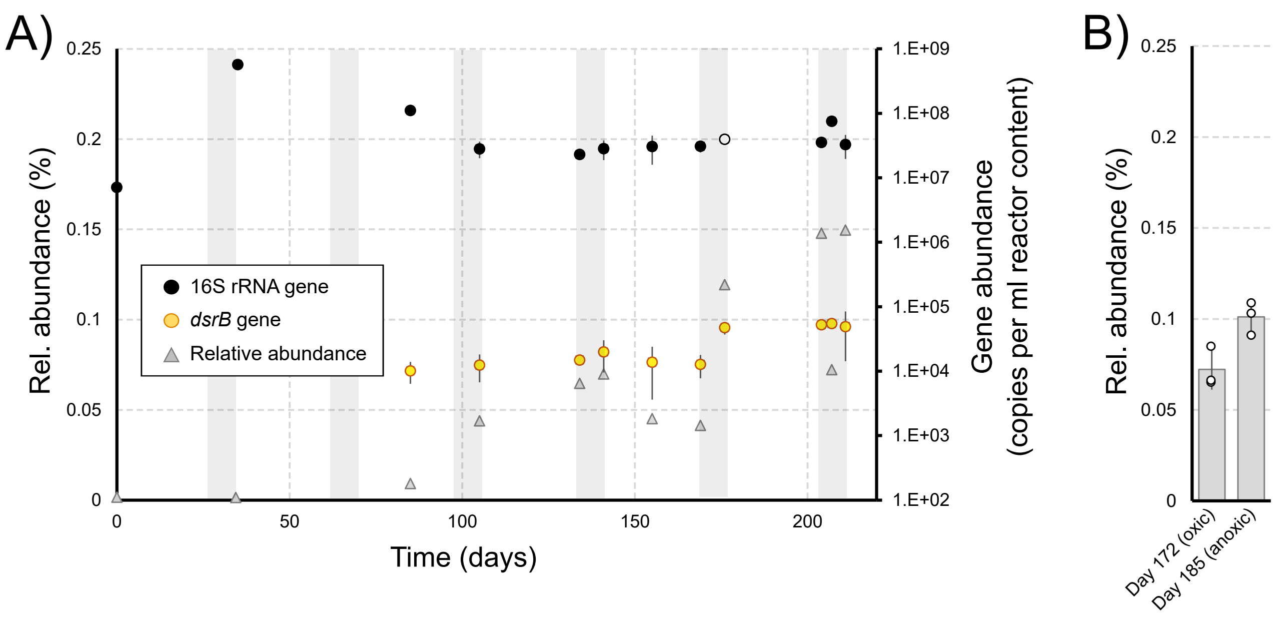


**Supplementary Figure S2.** Absolute and relative abundance of MAG CO124. Quantification of *dsrB* genes of MAG CO124 relative to total 16S rRNA genes of *Bacteria* and *Archaea* by quantitative PCR (A). Copy numbers of total 16S rRNA genes and *dsrB* genes are shown as mean ± SD. Except for one sample (open symbol) triplicates were used for qPCR analysis. Oxic periods are indicated by grey areas. Note that *dsrB* genes of MAG CO124 were below the limit of quantification in the inoculum and at day 35. For the timepoints of metagenome sequencing the relative abundance of MAG CO124 was estimated using metagenomic OTUs based on the single copy marker gene COG0202 (B). Relative mOTU abundance is shown as mean ± SD of three technical replicates. Source data are provided as Source Data file.

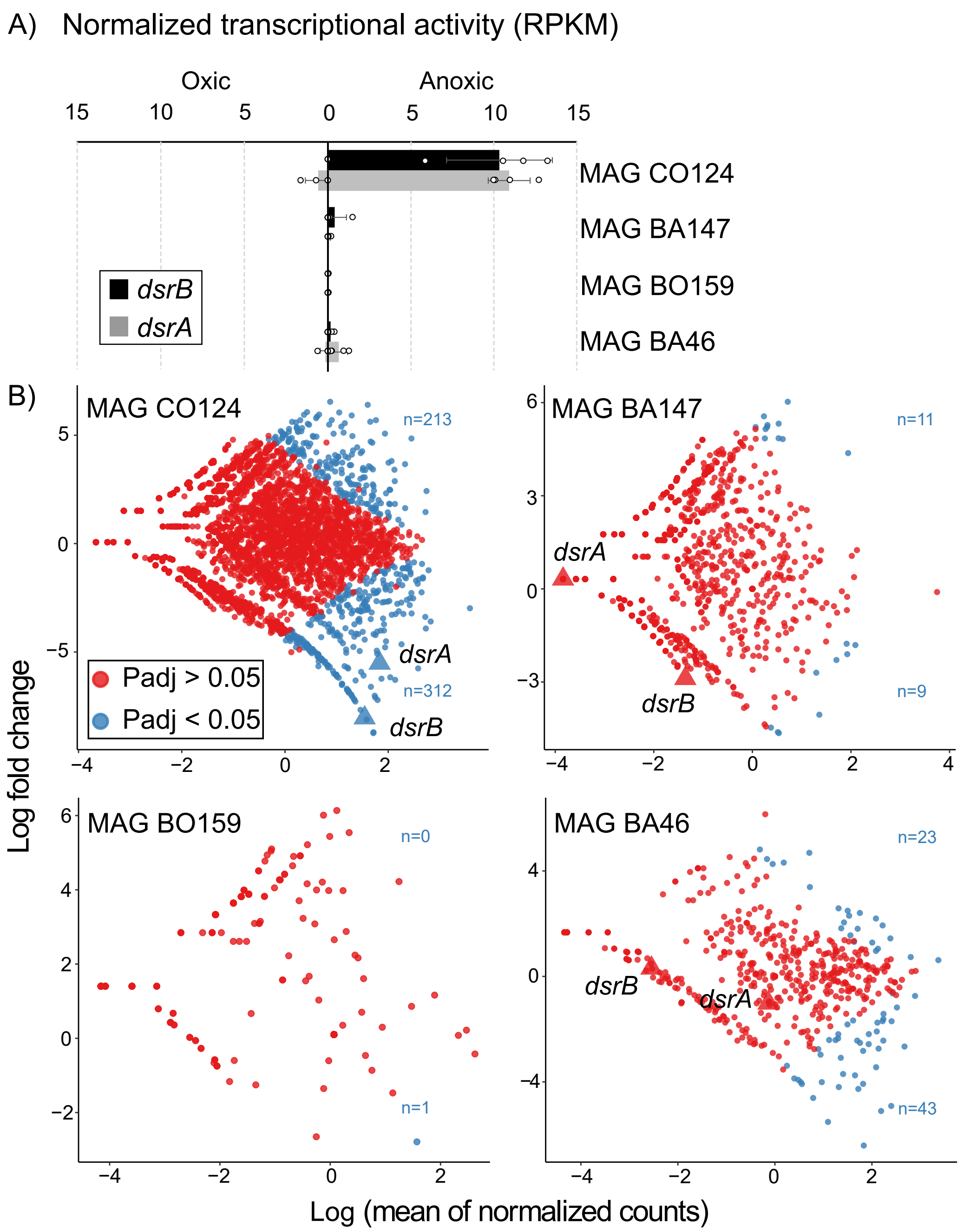


**Supplementary Figure S3.** Transcriptional activity of four *dsrAB*-containing *Acidobacteriota* MAGs. Normalized transcriptional activity (RPKM) of *dsrA* and *dsrB* genes under oxic and anoxic conditions (A). Bar charts show the mean ± SD of four technical replicates. Differentially expressed genes in four *dsrAB*-containing *Acidobacteriota* MAGs between oxic and anoxic conditions (B). Each dot in the plot indicate a transcriptionally active gene in the genome. Genes that significantly changed in their transcriptional levels according to the Wald test as implemented in DESeq2 (*P*-value adjusted < 0.05) were highlighted in blue, *dsrA* and *dsrB* genes are shown as triangles. *P*-values were corrected for multiple testing (Benjamini-Hochberg). Source data are provided as Source Data file.

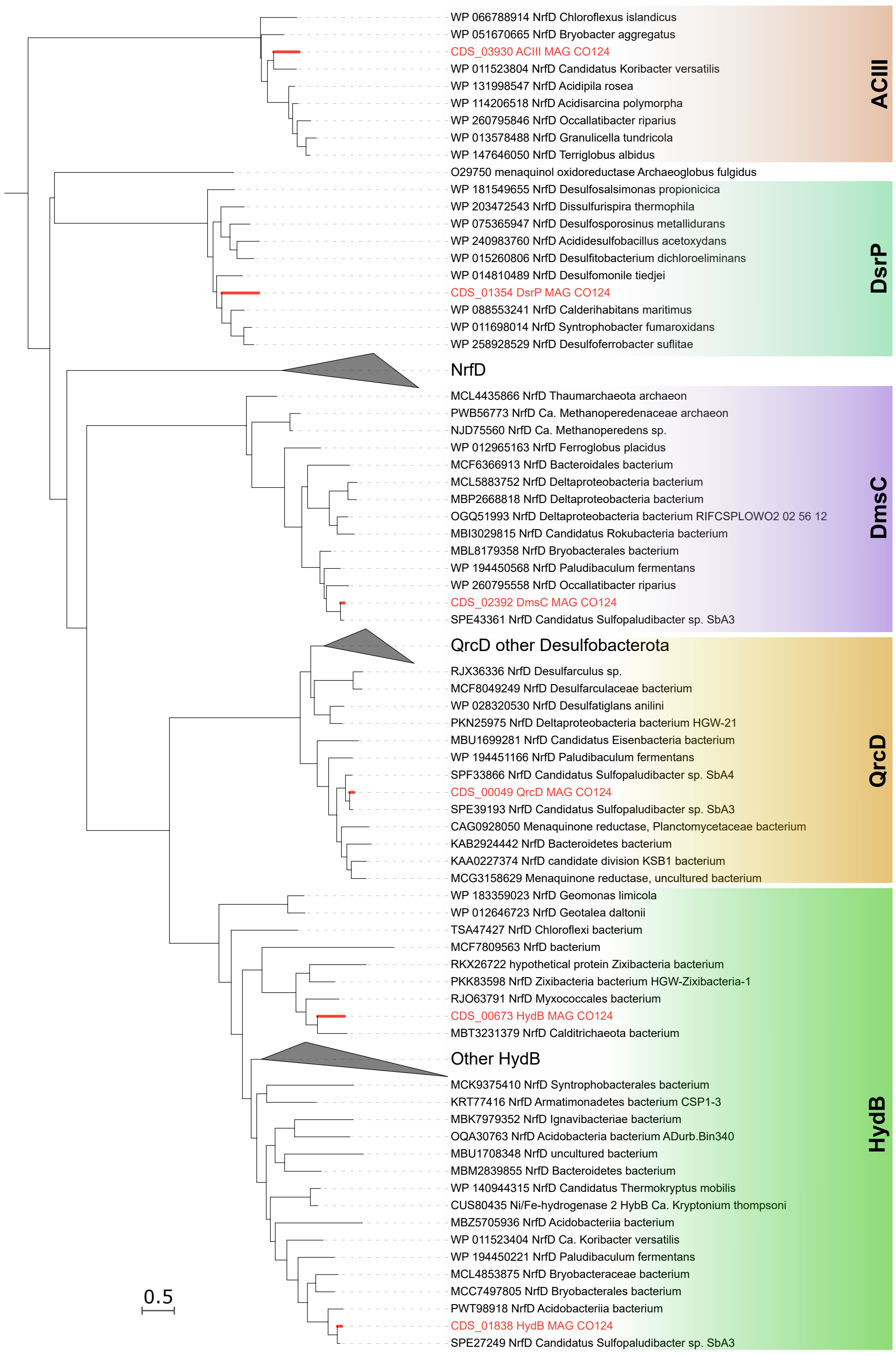


**Supplementary Figure S4.** Phylogenetic reconstruction of NrfD-like proteins. In total, 117 NrfD-like proteins were used for tree calculation using IQ-TREE 2 after automatic substitution model selection. NrfD-like proteins of MAG CO124 are shown in red. The tree represents the consensus of 1,000 ultrafast bootstrap analysis. ACIII, respiratory alternative complex ACIII; Dsr, dissimilatory sulfite reductase; Dms, dimethyl sulfoxide (DMSO) reductase; Qrc, quinone reductase complex; Hyb, group 2 NiFe hydrogenase.

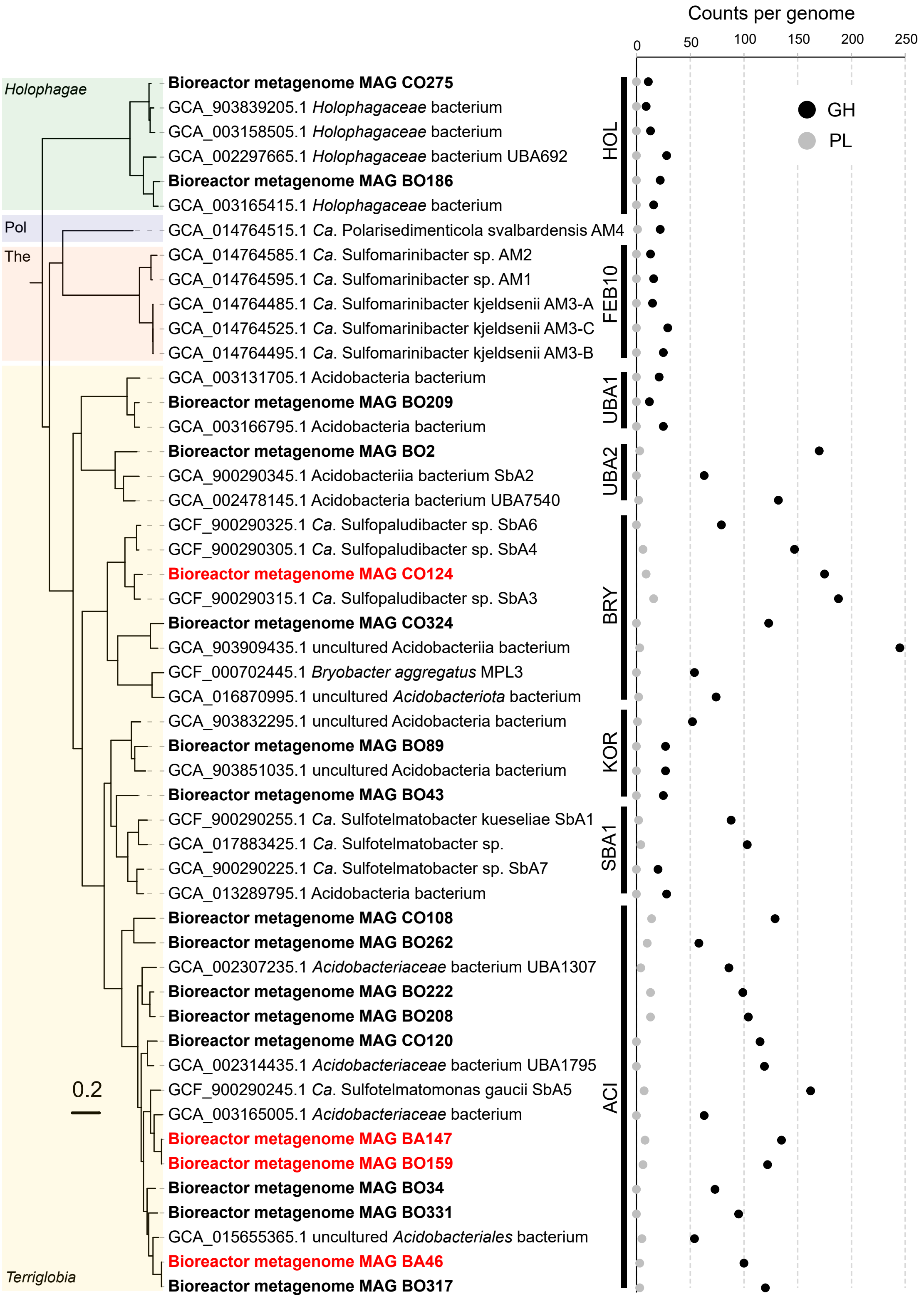


**Supplementary Figure S5.** Carbohydrate active enzymes in all *Acidobacteriota* MAGs shown in Figure 2. Displayed are counts per genome of glycoside hydrolases (GH) and polysaccharide lyases (PL), which are generally involved in carbohydrate degradation. The tree was adopted from Figure 2. Source data are provided as a Source Data file.
