# Supplementary material for: Oxygen respiration and polysaccharide degradation by a sulfate-reducing acidobacterium
Source: Nat Commun. 2023 Oct 10;14:6337. doi: 10.1038/s41467-023-42074-z (PMC10564751; doi:10.1038/s41467-023-42074-z)
Supplement: Supplementary file 3 — Description of Additional Supplementary Files [file 41467_2023_42074_MOESM3_ESM.pdf]

Description of Additional Supplementary Files:

Supplementary Data 1: Taxonomy, genome characteristics and coverage of MAGs recovered from the bioreactor.

Supplementary Data 2: Annotation and normalized transcriptional activity (reads per kilobase million, RPKM) of selected coding sequences (CDS) in MAG CO124, including genes that were potentially involved in sulfur metabolism. Differential expression of genes in response to oxic and anoxic conditions was tested for significance using the Wald test as implemented in DESeq2. P-values were corrected for multiple testing (Benjamini-Hochberg).

Supplementary Data 3: Annotation and normalized transcriptional activity (reads per kilobase million, RPKM) of genes encoding the aerobic respiratory chain in MAG CO124 (complex I-IV). The activity of C-III, AC-III and C-IV is also depicted in Figure 4. AC, alternative respiratory complex. Differential expression of genes in response to oxic and anoxic conditions was tested for significance using the Wald test as implemented in DESeq2. P-values were corrected for multiple testing (Benjamini-Hochberg).

Supplementary Data 4: Annotation and normalized transcriptional activity (reads per kilobase million, RPKM) of genes that were potentially involved in the degradation of pectin polysaccharides, including selected transporters, permeases and porins. Differential expression of genes in response to oxic and anoxic conditions was tested for significance using the Wald test as implemented in DESeq2. P-values were corrected for multiple testing (Benjamini-Hochberg).

Supplementary Data 5: Metagenomic assembly characteristics. Samples were taken at day 172 (oxic period) and day 185 (anoxic period) for metagenome sequencing. Triplicated metagenomic libraries were assembled together for each time point.

Supplementary Data 6: Annotation and normalized transcriptional activity (reads per kilobase million, RPKM) of all coding sequences (CDS) in MAG CO124. Differential expression of genes in response to oxic and anoxic conditions was tested for significance using the Wald test as implemented in DESeq2. P-values were corrected for multiple testing (Benjamini-Hochberg).

Supplementary Data 7: Potential function of genes shown in Figure 2.
