# Supplementary material for: Oxygen respiration and polysaccharide degradation by a sulfate-reducing acidobacterium
Source: Nat Commun. 2023 Oct 10;14:6337. doi: 10.1038/s41467-023-42074-z (PMC10564751; doi:10.1038/s41467-023-42074-z)
Supplement: Supplementary file 5 — Reporting Summary [file 41467_2023_42074_MOESM5_ESM.pdf]

nature portfolio

Corresponding author(s): Stefan Dyksma
Michael Pester

Last updated by author(s): Sep 5, 2023

Reporting Summary

Nature Portfolio wishes to improve the reproducibility of the work that we publish. This form provides structure for consistency and transparency in reporting. For further information on Nature Portfolio policies, see our Editorial Policies and the Editorial Policy Checklist.

Statistics

For all statistical analyses, confirm that the following items are present in the figure legend, table legend, main text, or Methods section.

n/a	Confirmed	
☐	☒	The exact sample size (*n*) for each experimental group/condition, given as a discrete number and unit of measurement
☐	☒	A statement on whether measurements were taken from distinct samples or whether the same sample was measured repeatedly
☐	☒	The statistical test(s) used AND whether they are one- or two-sided<br>*Only common tests should be described solely by name; describe more complex techniques in the Methods section.*
☒	☐	A description of all covariates tested
☐	☒	A description of any assumptions or corrections, such as tests of normality and adjustment for multiple comparisons
☐	☒	A full description of the statistical parameters including central tendency (e.g. means) or other basic estimates (e.g. regression coefficient) AND variation (e.g. standard deviation) or associated estimates of uncertainty (e.g. confidence intervals)
☐	☒	For null hypothesis testing, the test statistic (e.g. *F*, *t*, *r*) with confidence intervals, effect sizes, degrees of freedom and *P* value noted<br>*Give P values as exact values whenever suitable.*
☒	☐	For Bayesian analysis, information on the choice of priors and Markov chain Monte Carlo settings
☒	☐	For hierarchical and complex designs, identification of the appropriate level for tests and full reporting of outcomes
☒	☐	Estimates of effect sizes (e.g. Cohen's *d*, Pearson's *r*), indicating how they were calculated

Our web collection on statistics for biologists contains articles on many of the points above.

Software and code

Policy information about availability of computer code

Data collection	No software was used for data collection
Data analysis	DADA2 pipeline (version 2022.2.0), Qiime2 (version 2022.2.1), SINA classifier and the SILVA SSU reference database (version 138.1), PAST (version 3.24), BBTools (version 38.22), MEGAHIT (version 1.2.9), Bowtie2 (version 2.3.5.1), MetaBAT2 (version 2.12.1), MaxBin2 (version 2.2.7), MetaCoAG (version 1.0), DAS_Tool (version 1.1.4), CheckM (version 1.0.7), GTDB-Tk (version 2.1.1), bbmap.sh (BBMap version 38.22), SPAdes (version 3.14.0), METABOLIC-C (version 4.0), MetaErg (version 1.2.0), DiSCo (version 1.0.0), dbCAN2, IQ-TREE 2 (version 2.2.0.3), RiboDetector (version 0.2.6) and DESeq2 (version 1.41.8).

For manuscripts utilizing custom algorithms or software that are central to the research but not yet described in published literature, software must be made available to editors and reviewers. We strongly encourage code deposition in a community repository (e.g. GitHub). See the Nature Portfolio guidelines for submitting code & software for further information.

Data

Policy information about availability of data

All manuscripts must include a data availability statement. This statement should provide the following information, where applicable:

- Accession codes, unique identifiers, or web links for publicly available datasets
- A description of any restrictions on data availability
- For clinical datasets or third party data, please ensure that the statement adheres to our policy

Amplicon sequences from the 16S rRNA gene survey were deposited in NCBI BioProject PRJNA923133. Metagenomes, metatranscriptomes, and MAGs are available

under NCBI BioProject PRJNA923161. Reference genomes and proteins for phylogenetic reconstructions were obtainded from GenBank and the SWISS-PROT database, respectively. All accession numbers used for tree calculation are displayed in the respective figures (Figure 2, Supplementary Figures S4 and S5).

Research involving human participants, their data, or biological material

Policy information about studies with human participants or human data. See also policy information about sex, gender (identity/presentation), and sexual orientation and race, ethnicity and racism.

Reporting on sex and gender	n/a
Reporting on race, ethnicity, or other socially relevant groupings	n/a
Population characteristics	n/a
Recruitment	n/a
Ethics oversight	n/a

Note that full information on the approval of the study protocol must also be provided in the manuscript.

Field-specific reporting

Please select the one below that is the best fit for your research. If you are not sure, read the appropriate sections before making your selection.

☐ Life sciences ☐ Behavioural & social sciences ☒ Ecological, evolutionary & environmental sciences

For a reference copy of the document with all sections, see nature.com/documents/nr-reporting-summary-flat.pdf

Ecological, evolutionary & environmental sciences study design

All studies must disclose on these points even when the disclosure is negative.

Study description	To study the ecophysiology of sulfur-dissimilating Acidobacteriota, we followed sulfur-cycling in a bioreactor that was operated under fluctuating oxygen regimes with a periodical switch between oxic conditions (50% air-O2 saturation) for one week and anoxic/sulfate-reducing conditions for four weeks over a period of more than 200 days. 16S rRNA gene amplicon sequencing, quantitative PCR, metagenomics and metatrascriptomics were combined to determine the microbial community composition and their function. DNA or RNA were extracted from 3 to 4 replicates. Technical replicates of metagenomes (n=3) and metatranscriptomes (n=4) were both recovered at day 172 and day 185.
Research sample	A bioreactor was inoculated with peat soil from an acidic fen (pH 4-5), an environment that is dominated by Acidobacteriota, shows active sulfur cycling, and is characterized by periods of oxic and anoxic conditions in combination with an acidic pH as a selective pressure that discriminates against the majority of cultured sulfate-reducing microorganisms.
Sampling strategy	No statistics were used to decide on the samples size. Metagenomics and metatranscriptomics was performed in three and four technical replicates, respectively. 16S rRNA gene amplicon sequencing, qPCR analyses and analytical measurements were performed in three technical replicates. The operating volume of the bioreactor (1 liter) was restricting the number of samples that could be taken over time.
Data collection	Analytical measurements were done by S. Dyksma. Metagenomes and -transcriptomes were sequenced on an Illumina NextSeq2000 sequencer at the Leibniz Institute DSMZ, raw data is stored on the DSMZ server and multiple hard drives. Amplicon sequencing was performed on an Illumina MiSeq at the Leibniz Institute DSMZ.
Timing and spatial scale	Peat soil was sampled on 2021-09-24. The bioreactor was started on 2021-09-27 and samples were taken over a time period of 211 days. Samples for amplicon sequencing were taken on days 0 (inoculum), 28, 35, 49, 64, 71, 85, 98, 105, 113, 134, 141, 155, 169, 172, 176, 185, 204 and day 211. Samples were taken at least before and after each oxic period to follow changes in the microbial community composition. Samples for metagenome and metatranscriptome sequencing were taken on day 172 (oxic period) and day 185 (anoxic period) with active sulfate production and sulfate reduction, respectively.
Data exclusions	For qPCR analyses of day 172 and day 185, copy numbers of total 16S rRNA genes could not be determined or provided unreliable results because DNA extracts were already used up for other analyses (metagenomics and dsrB qPCR analyses) and could not be repeated. To compensate for these missing time points, the relative abundance of Acidobacteriota MAG CO124 was determined by the PCR-independent metagenomic OTU approach.
Reproducibility	The long-term enrichment cultivation is a continuous process, embedded in a complex bioreactor setup. Therefore, enrichment cultivation was so far not reproduced with starting material sampled from the same field site. Samples were analyzed in technical repicates. Replicates from each experiment were in the same range.
Randomization	All samples were taken randomly from the bioreactor after thorough mixing of the reactor content.

Blinding	Blinding was not relevant in this study, as we worked with environmental samples and not with animals or humans.

Did the study involve field work? ☒ Yes ☐ No

Field work, collection and transport

Field conditions	Peat soil was sampled on 2021-09-24 irrespective of the temperature and rainfall. A detailed description of the long-term experimental field site “Schlöppnerbrunnen II” was addressed in several earlier publications.
Location	Soil was sampled from the acidic fen “Schlöppnerbrunnen II” located in south-eastern Germany (50°08’38"N, 11°51’41"E)
Access & import/export	Sampling was conducted at the long-term experimental field site “Schlöppnerbrunnen II” with no permits required for sampling.
Disturbance	To reduce ecosystem disturbance, sampling was restricted to the long-term experimental field site “Schlöppnerbrunnen II”. A small volume (1 l) of peat soil was sampled from the surface layer.

Reporting for specific materials, systems and methods

We require information from authors about some types of materials, experimental systems and methods used in many studies. Here, indicate whether each material, system or method listed is relevant to your study. If you are not sure if a list item applies to your research, read the appropriate section before selecting a response.

Materials & experimental systems

n/a	Involved in the study
☒	☐ Antibodies
☒	☐ Eukaryotic cell lines
☒	☐ Palaeontology and archaeology
☒	☐ Animals and other organisms
☒	☐ Clinical data
☒	☐ Dual use research of concern
☒	☐ Plants

Methods

n/a	Involved in the study
☒	☐ ChIP-seq
☒	☐ Flow cytometry
☒	☐ MRI-based neuroimaging
